# Laser‐facilitated epicutaneous immunotherapy with hypoallergenic beta‐glucan neoglycoconjugates suppresses lung inflammation and avoids local side effects in a mouse model of allergic asthma

**DOI:** 10.1111/all.14481

**Published:** 2020-07-16

**Authors:** Evgeniia Korotchenko, Viktoria Schießl, Sandra Scheiblhofer, Mario Schubert, Elfriede Dall, Isabella A. Joubert, Helen Strandt, Theresa Neuper, Muamera Sarajlic, Renate Bauer, Mark Geppert, David Joedicke, Sabrina Wildner, Susanne Schaller, Stephan Winkler, Gabriele Gadermaier, Jutta Horejs‐Hoeck, Richard Weiss

**Affiliations:** ^1^ Department of Biosciences University of Salzburg Salzburg Austria; ^2^ Research and Development University of Applied Biosciences Upper Austria Hagenberg Austria

**Keywords:** dendritic cell targeting, epicutaneous immunotherapy, glycoconjugates, laser, skin vaccination

## Abstract

**Background:**

Allergen‐specific immunotherapy via the skin targets a tissue rich in antigen‐presenting cells, but can be associated with local and systemic side effects. Allergen‐polysaccharide neoglycogonjugates increase immunization efficacy by targeting and activating dendritic cells via C‐type lectin receptors and reduce side effects.

**Objective:**

We investigated the immunogenicity, allergenicity, and therapeutic efficacy of laminarin‐ovalbumin neoglycoconjugates (LamOVA).

**Methods:**

The biological activity of LamOVA was characterized in vitro using bone marrow‐derived dendritic cells. Immunogenicity and therapeutic efficacy were analyzed in BALB/c mice. Epicutaneous immunotherapy (EPIT) was performed using fractional infrared laser ablation to generate micropores in the skin, and the effects of LamOVA on blocking IgG, IgE, cellular composition of BAL, lung, and spleen, lung function, and T‐cell polarization were assessed.

**Results:**

Conjugation of laminarin to ovalbumin reduced its IgE binding capacity fivefold and increased its immunogenicity threefold in terms of IgG generation. EPIT with LamOVA induced significantly higher IgG levels than OVA, matching the levels induced by s.c. injection of OVA/alum (SCIT). EPIT was equally effective as SCIT in terms of blocking IgG induction and suppression of lung inflammation and airway hyperresponsiveness, but SCIT was associated with higher levels of therapy‐induced IgE and TH2 cytokines. EPIT with LamOVA induced significantly lower local skin reactions during therapy compared to unconjugated OVA.

**Conclusion:**

Conjugation of ovalbumin to laminarin increased its immunogenicity while at the same time reducing local side effects. LamOVA EPIT via laser‐generated micropores is safe and equally effective compared to SCIT with alum, without the need for adjuvant.

## Graphical abstract

Epicutaneous immunotherapy (EPIT) targets a tissue rich in antigen‐presenting cells. Laser‐facilitated EPIT combines controlled and efficient barrier disruption with a laser‐induced adjuvant effect. However, application of native allergens to the skin can induce unwanted side effects. Coupling the β‐glucan laminarin to allergens can markedly reduce their IgE binding capacity. β‐glucan neoglycoconjugates efficiently target dendritic cells via the C‐type lectin receptor dectin‐1, boosting their immunogenicity. In a mouse model of allergic asthma, laminarin‐ovalbumin conjugates (LamOVA) diminished local side effects in vivo, while inducing higher levels of blocking IgG compared with uncoupled OVA. EPIT with LamOVA was equally efficient in improving lung functions compared to classical SCIT. Abbreviations: EPIT, epicutaneous immunotherapy; LamOVA, laminarin‐ovalbumin conjugates; OVA, ovalbumin; PBS, phosphate‐buffered saline; Penh, resistance and compliance data are shown as area under the curve (AUC) of a methacholine challenge dose response curve; SCIT, subcutaneous immunotherapy

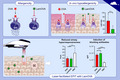

**Abbreviations**
EPIT: epicutaneous immunotherapy
LamOVA: laminarin‐ovalbumin conjugates
OVA: ovalbumin
PBS: phosphate buffered saline
Penh: resistance and compliance data are shown as area under the curve (AUC) of a methacholine challenge dose response curve
SCIT: subcutaneous immunotherapy

## 1 INTRODUCTION

Epicutaneous allergen‐specific immunotherapy (EPIT) has been introduced almost 100 years ago and only recently revisited.^1^ Current studies show that EPIT can lead to a potent reduction of allergic symptoms and that the efficacy of the therapy is dependent on the allergen dose and the pretreatment of the skin. However, depending on the degree of barrier disruption, epicutaneous application of allergen can induce local and even systemic side effects in some individuals.^2^ Alternatively, therapy has been performed by application of occlusive patches to intact skin leading to epicutaneous absorption due to maceration.^3^ The major pitfall of this method is the inefficient uptake, requiring numerous applications of high doses.^4^

Due to the high number of antigen‐presenting cells (APCs) in the epidermis and dermis, skin‐based immunization often requires smaller amounts of antigen to induce immune responses compared to subcutaneous (s.c.) or intramuscular injections.^5^ APCs express a large variety of pattern‐recognition receptors (PRRs) determining their main function, that is, sensing of pathogens and induction of immune reactions.^6^ One group of PRRs are C‐type lectin receptors (CTLs), which bind to carbohydrates in a calcium‐dependent manner. Together with other receptors, stimulation of CTLs determines the activation status of dendritic cells (DCs) and subsequent T‐cell polarization.^7^ Therefore, the use of protein neoglycoconjugates for immunizations can increase vaccine potency.

As a prominent member of the CTL family, Dectin‐1 is functionally equivalent in mice and humans.^8^ It recognizes β‐glucans and some proteins such as tropomyosin,^9^ and upon ligand binding activates innate immune responses. Murine Dectin‐1 is expressed on macrophages, neutrophils, and dermal DCs.^10^ In humans, Dectin‐1 is not restricted to cells of the myeloid lineage but can also be found on epithelial cells,^8^, ^11^, ^12^ keratinocytes,^13^ B cells, and subpopulations of T cells.^8^ As Dectin‐1 is expressed on dermal DCs, its use as a vaccination platform for targeted skin vaccination is highly promising. Here, we used 4‐5 kDa laminarin/ovalbumin (LamOVA) conjugates for allergen‐specific immunotherapy (AIT). Laminarin has strong immunostimulatory properties and has been employed in tumor therapy for activation of the innate immune system.^14^ Moreover, polysaccharides can mask epitopes on allergens preventing IgE cross‐linking on mast cells and histamine release, decreasing the risk of side effects.^15^

Skin has a protective function, and its outermost layer, the stratum corneum, represents a tight barrier that has to be overcome for antigen delivery. We use infrared laser to form micropores in the upper layers of the skin.^16^ Besides facilitating vaccine penetration, the local tissue damage caused by laser treatment attracts large numbers of APCs and creates a pro‐inflammatory milieu, thereby acting as a physical adjuvant for skin vaccinations.^17^

In our current work, we evaluated the immunostimulatory capacity of LamOVA conjugates and their efficacy in a preventive and therapeutic BALB/c mouse model of allergic lung inflammation. Laser‐facilitated EPIT with LamOVA significantly reduced airway hyperresponsiveness and local side effects in vivo.

## 2 MATERIALS AND METHODS

A more detailed version of materials and methods and a complete list of used antibodies and their dilutions (Table S1) can be found in the Supplementary Materials.

### 2.1 Generation and analysis of allergen‐carbohydrate neoglycoconjugates

Laminarin from laminaria digitata (Sigma, L9634, batch SLBP4829V) was dialyzed with a cut‐off of 2 kDa to remove low molecular weight impurities and partially oxidized with sodium (meta)periodate (Sigma).^15^ Briefly, 400 mg of laminarin were reacted with 95.2 mg sodium (meta)periodate for 1 hour at RT followed by dialysis against 50 mM phosphate buffer, pH 7. Laminarin and its oxidized variant were analyzed by two‐dimensional NMR spectroscopy. Oxidized laminarin was coupled to endotoxin‐free ovalbumin (OVA, EndoFit™ Ovalbumin, InvivoGen) using 2‐step reductive amination with 50 mM sodium cyanoborohydride (NaCNBH_3_) and 10 mM sodium borohydride (NaBH_4_) as previously described.^15^ Resulting neoglycoconjugates were separated by size‐exclusion chromatography on a HiPrep 26/100 Sephacryl® S‐200 HR column (GE Healthcare Life Sciences), and fractions were analyzed by 10% SDS‐PAGE. Fractions containing high molecular weight (>70 kDa) laminarin‐ovalbumin conjugates (LamOVA) were used for further experiments.

Carbohydrate concentration in conjugates was estimated using anthrone method ^18^ and OVA concentration was determined by amino acid analysis.^19^ The hydrodynamic radius of conjugates was analyzed by dynamic light scattering using a Zetasizer Nano ZS with a DTS1070 capillary cell (Malvern Instruments).

Biological activity of laminarin in LamOVA conjugates was analyzed by ELISA using a soluble murine Fc‐Dectin‐1a receptor (InvivoGen) and by Microscale Thermophoresis (MST) using a Monolith NT.115 RED instrument (Nanotemper, Munich, Germany). Hypoallergenicity was analyzed in vitro as a measure of rat basophil leukemia cell (RBL‐2H3) activation by β‐hexosaminidase release assay.^15^

### 2.2 Uptake and activation analysis using bone marrow‐derived dendritic cells

Bone marrow‐derived dendritic cells (BMDCs) were harvested from mouse femur and tibia and incubated with either 20 ng/mL murine GM‐CSF (Immunotools, Cat. Nr 12343125) or 200 ng/mL human Flt3‐L (AcroBiosystems, Cat. Nr FLL‐H55H7) as described^20^, ^21^ with minor changes.

LamOVA effects on CD86 and Dectin‐1 expression on CD11c + Flt3‐L—generated DCs (FL‐DCs) and on CD11c + MHCII^high^ CD11b^int^ GM‐CSF—derived DCs (GM‐DCs) were assessed. Activation of OVA‐specific naïve DO11.10 T cells was analyzed in co‐culture assays. BMDC activation, T‐cell proliferation, and uptake were assessed by flow cytometry as described in Supplementary Materials. Cytokine concentration in supernatants was analyzed by LEGENDplex assay (Biolegend) using the 13‐plex mouse inflammation panel and the 13‐plex mouse proinflammatory chemokine panel according to the manufacturer's instructions.

### 2.3 Animal experiments

Female BALB/c mice aged 6‐8 weeks were obtained from Janvier (Le Genest‐Saint‐Isle, France) and maintained at the animal facility of the University of Salzburg in an SPF environment according to local guidelines. All animal experiments were conducted in compliance with EU Directive 2010/63/EU and have been approved by the Austrian Ministry of Education, Science and Research, permit number BMBWF‐66.012/0006‐V/3b/2018. Blood samples were drawn on days 27, 35, 55 (prevention) and 16 and 86 (therapy) by puncture of v. saphena.

To study immunogenicity of the conjugates, mice (n = 6) were immunized three times with 20 µg of OVA, LamOVA conjugates (20 µg OVA coupled to 49 µg laminarin), a mixture of equivalent amounts of OVA + laminarin, or laminarin and PBS (sham controls). The antigens were applied to laser‐microporated skin on days 1, 15, and 29. Microporation was performed using a PLEASE® professional infrared laser device (Pantec Biosolutions, Ruggell, Liechtenstein) with a total fluence of 8.3 J/cm^2^ as previously described.^15^ After the third immunization, mice were sensitized two times in a ten‐day interval (days 36 and 46) by i.p. injections of 1 µg EndoFit™ OVA adsorbed to 50% (v/v) alum (Alu‐Gel‐S, Serva) in endotoxin‐free PBS. To induce lung inflammation, animals were challenged by exposure to aerosolized grade V OVA from Sigma (A5503) that has been purified from endotoxin using Triton‐X114 method^22^ (10 mg/mL in 0.9% NaCl, LPS <0.39 pg per 1 µg OVA), for 30 minutes on days 53‐55 using a Pariboy SX jet nebulizer with a Pari LL nebulizer head (Pari GmbH).

To study therapeutic efficacy, animals (n = 12) were sensitized by two i.p. injections with 10 µg of EndoFit™ OVA/alum on days 1 and 8. On day 15, mice were challenged intranasally with 10 µg of EndoFit™ OVA in 40 µL PBS, under isoflurane anesthesia. One day after the challenge, lung function and specific serum IgE were analyzed to confirm that all animals in the treatment groups had an asthma‐like phenotype and OVA‐specific IgE. Eight therapeutic immunizations were performed between days 18 and 67 in weekly intervals. Mice were treated with LamOVA conjugates, OVA, laminarin, or PBS. 35 µg of OVA and/or 86 µg of laminarin equivalents were applied on 1 cm^2^ of laser‐microporated skin. As a positive control, s.c. injections with 35 µg EndoFit™ OVA/alum were performed. On days 84‐86, mice were challenged with OVA aerosol.

### 2.4 Analysis of local side effects

For analysis of erythema severity, mice were photographed on days 47 or 54 with a SONY ILCE‐7M2 camera, 28‐70 mm zoom lens with a constant source of light using.JPG format. MATLAB R2019a was used for analysis. Briefly, the area of laser microporation (region of interest, ROI) was defined by an investigator blinded to the treatment. Based on the gray value of the selected ROI, a threshold was manually selected, to define the erythema that was darker than healthy skin. Erythema size and mean blob size were calculated from the selected ROI. Erythema size is calculated as the ratio of pixels above the chosen threshold to the number of pixels inside the ROI. A blob is defined as the sum of directly connected pixels of the segmented erythema. The mean value of the five biggest blobs was evaluated.

### 2.5 Lung function analysis

The day after the aerosol challenge, lung function was analyzed by whole‐body plethysmography (WBP) using a Buxco 6‐chamber unrestrained WBP system (Data Sciences International). The next day, lung resistance/compliance measurement was performed using a FinePointe Series Resistance and Compliance (RC) site (Data Sciences International). Bronchoalveolar lavage fluid (BALF) was collected, and its cellular composition was analyzed by flow cytometry staining for Siglec F, CD45, CD4, CD8, CD19, and Ly6G/Ly6C (Gr‐1).

### 2.6 Lung digestion

Lung tissue was digested, and total leukocytes were separated using biotinylated CD45 and BD™ IMag Streptavidin Particles (BD Biosciences). Cells were stained for CD11b, CD24, CD4, CD45, Ly6G/Ly6C (Gr‐1), MHC‐II, and Siglec F and analyzed by flow cytometry.

### 2.7 Analysis of OVA‐specific serum IgG/IgE and cell‐bound IgE

OVA‐specific IgG isotypes in sera were analyzed by direct ELISA. OVA‐specific serum IgE was measured using a rat basophil leukemia cell (RBL‐2H3) assay. Briefly, cells were incubated for 2 hours with mouse sera diluted 1:150 in medium, followed by three washing steps and 1 hour stimulation with 0.3 µg/mL EndoFit™ OVA. β‐hexosaminidase levels in supernatants were measured and degranulation was calculated as percentage of total lysis from control wells lysed with Triton X‐100.

Cell‐bound IgE was analyzed ex vivo by basophil activation test from whole blood drawn one day after the second aerosol challenge. Cells were stimulated in the presence or absence of autologous serum with 2 ng/mL OVA for 2 hours at 37°C, 5% CO_2_, 95% humidity. After the incubation, cells were stained for IgE, CD4, CD19, and CD200R and analyzed by flow cytometry on a FACS Canto II flow cytometer (BD Biosciences).

### 2.8 Lymphocyte restimulation

Spleens were harvested for T‐cell restimulation with OVA. After erythrocyte lysis in Ammonium‐Chloride‐Potassium (ACK) lysing buffer, splenocytes were resuspended in T‐cell medium (RPMI‐1640, 10% FCS, 25 mM Hepes, 2 mM L‐Glu, 100 µg/mL streptomycin, 100 U/mL penicillin) and 0.6 × 10^6^ cells/well were stimulated with 0.1 mg/mL EndoFit™ OVA and incubated for 3 days at 37°C, 5% CO_2_. Supernatants were harvested and analyzed by LEGENDplex immunoassay using the mouse T helper 13‐plex cytokine panel. Cells were stained for CD4, CD25, CD44, FoxP3, GATA3 and analyzed by flow cytometry on a Cytoflex S flow cytometer (Beckman Coulter).

### 2.9 Statistical analysis

Data were statistically analyzed using GraphPad Prism 7 using one‐way ANOVA with Tukey's post hoc test unless otherwise specified. *P*‐values are shown as: *P ≤ .05, **P ≤ .01, ***P ≤ .001, ****P ≤ .0001.

## 3 RESULTS

### 3.1 Generation of laminarin‐OVA conjugates

**Figure 1:**
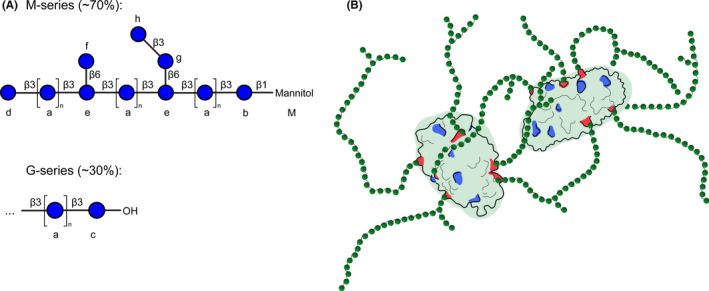
(A) Structure of laminarin as determined by NMR. Glucose residues that are distinguishable by NMR spectroscopy are labeled with small letters a to h and the mannitol is abbreviated with M. β1‐3 and β1‐6 glycosidic bonds are indicated as β3 and β6, respectively. (B) Schematic model depicting OVA (PDB: 1OVA) with 6 covalently linked chains of laminarin. The six lysine residues that have a fractional accessible surface area >0.5 at least in one of the four structures in the asymmetric unit of the crystal structure are shown in red, the other lysines in blue. A potential cross‐linking of two OVA molecules is shown

NMR analysis of the used laminarin batch (SLBP4829V) indicated approx. 70% M‐series (terminated with 1‐linked d‐mannitol) and 30% G‐series (containing only glucose) in the fresh and also in the dialyzed laminarin sample (Figure 1A and Figures S1 and S2). Approx. 1.5 branch sites per molecule were observed. These findings are in agreement with previously published data (Table S2).^23^, ^24^ Previous studies have found laminarin from the same supplier to be in the size range of 4‐5 kD^25^ which is in line with data from Read et al^24^ and also fits to our NMR data. For all further calculations, we thus estimated a MW of 4.5kD for the used laminarin batch. Upon treatment with periodate, all the signals of the mannitol (Figure 1A, Figures S1 and S2, unit M) and the unprotected glucose moieties at the reducing end (unit c) disappeared and the terminal glucose moieties (units d, f and h) decreased in their signal intensity, indicating successful oxidation. These were also the expected sites for periodate oxidation, indicated in red in Figure S2, as they contain accessible and neighboring OH groups that can react with periodate to generate reactive aldehyde groups. This is in agreement with the study by Read et al, who showed by mass spectrometry that only the terminal Glc moieties of both, the main chain and the side chains are oxidized.^24^

Neoglycoconjugates were generated by coupling of oxidized laminarin to EndoFit™ OVA by reductive amination. Size‐exclusion chromatography (Figure S3A) was used to separate neoglycoconjugates by size, followed by SDS‐PAGE of the fractions (Figure S3B). Based on visual analysis, fractions migrating above 70 kDa were pooled (LamOVA) and further analyzed.

To determine OVA concentration in conjugates, amino acid analysis was used (Figure S3C), as common methods of protein quantification such as UV absorption, Bradford assay, or BCA assay are all affected by the carbohydrate moiety. Additionally, this method allows estimating the amount of lysine residues which participated in reductive amination. For LamOVA, an average of 6 out of 20 lysines was coupled to laminarin. This is in line with in silico prediction using the VADAR server,^26^ showing that 6 lysines have a fractional accessible surface area (ASA) >0.5 at least in one of the four structures in the asymmetric unit of the crystal structure 1OVA.pdb. These are K39, K78, K105, K135, K236, and K292 (shown in red in Figure 1B), which are therefore most likely involved in laminarin coupling. This coupling rate of 6 molecules laminarin to 1 molecule of OVA corresponds to a carbohydrate to protein ratio of 0.6 (w/w). This was supported by SDS‐PAGE where the majority of the conjugates migrated between 70 and 130 kD. As laminarin does not bind SDS, the net charge‐to‐mass ratio of the protein‐SDS complex is reduced, resulting in an overestimation of its molecular weight. Thus, we surmise that the majority of LamOVA consists of monomeric OVA with an average of 6 molecules of laminarin covalently linked (Figure 1B). An additional faint band migrating above 180 kD in the SDS‐PAGE in the high molecular weight fractions 26‐29 probably corresponds to conjugates containing more than one molecule of OVA due to cross‐linking via laminarin. As shown by dynamic light scattering (Figure S3D), conjugates were homogeneous in size and had a diameter of 8.27 ± 0.75 nm, which also supports the prevalence of monomeric rather than cross‐linked LamOVA conjugates (given a hydrodynamic radius of ~3 nm for monomeric OVA).

Estimation of carbohydrate content in LamOVA by the anthrone method indicated a higher carbohydrate/protein ratio of 2.5 (w/w), which can be explained by the inaccuracy of the method, the presence of noncovalently attached laminarin and/or free laminarin not completely removed by SEC. Nevertheless, we calculated with a carbohydrate/protein ratio of 2.5 when using laminarin controls in the subsequent experiments to avoid any potential underestimation of the amount of laminarin in the conjugates.

### 3.2 Laminarin is biologically active after conjugation

**Figure 2:**
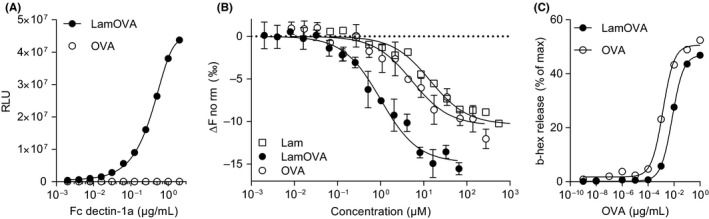
Binding of soluble Dectin‐1a receptor (fused to IgG‐Fc) to plate‐bound LamOVA or OVA (A) or soluble LamOVA, OVA, and laminarin (B). Data are shown as relative light units (RLU) of a luminometric ELISA (A) or as changes in normalized fluorescence (ΔFnorm) of MST measurements (mean ± SD of three independent binding experiments). (C) Stimulation of IgE‐loaded RBL cells with LamOVA or OVA. Basophil IgE‐mediated degranulation is presented as percentage of total beta‐hexosaminidase (b‐hex) release

To ensure that the structure of laminarin was not destroyed during mild periodate oxidation and that the polysaccharide remained biologically active after coupling, binding to Dectin‐1 was assessed by ELISA and MST. As shown in Figure 2A, plate‐bound LamOVA was recognized by Dectin‐1, whereas no binding to unconjugated OVA was observed. To verify that the observed differences were not due to relative coating efficiencies of OVA and LamOVA or unspecific binding of LamOVA due to denaturation and aggregation, we confirmed these results in solution using MST (Figure 2B) and included an unspecific soluble receptor (TLR5) as a control (Figure S4). Using Hill fit model for multiple binding, the apparent dissociation constant (Kd_app_) for binding of LamOVA and 4.5 kD laminarin to Dectin‐1 was 0.99 ± 0.17 µM vs 13.98 ± 3.03 µM (calculating with a carbohydrate to protein ratio of 0.6). Even considering additional noncovalently linked laminarin in the conjugate preparation (carbohydrate to protein ratio of 2.5 based on anthrone method), the resulting Kd_app_ value of 4.22 ± 0.72 µM was still threefold lower compared with 4.5 kD laminarin. These data indicate that LamOVA conjugates bind Dectin‐1 in solution with a threefold to 13‐fold higher affinity compared to unconjugated laminarin. Similar values were obtained for oxidized laminarin (data not shown). Based on the OVA concentration in the conjugates, coupling of OVA to laminarin increased its affinity for Dectin‐1 30‐fold compared to soluble OVA (0.17 ± 0.03 µM vs 5.33 ± 1.75 µM). Although LamOVA showed a higher binding affinity for TLR5 than laminarin (6 ± 2 µM vs 74 ± 46 µM; Figure S4), this was still sixfold lower compared with its affinity for Dectin‐1, confirming the specificity. The partial binding of LamOVA to TLR‐5 was probably due to the OVA moiety of the conjugates, as unconjugated OVA showed the highest affinity for TLR‐5 (0.8 ± 0.3 µM).

### 3.3 LamOVA conjugates activate RBL cells to a fivefold lesser extent than unmodified OVA

Hypoallergenicity is a prerequisite for AIT via the skin, as administration of native allergen to barrier disrupted skin has been shown to induce local or even systemic side effects.^2^ We hypothesized that laminarin polysaccharide chains (6 kDa) would mask IgE epitopes of OVA and prevent mast cell and basophil degranulation in vivo. To prove that conjugates were less allergenic than unconjugated OVA, rat basophil leukemia (RBL) cells were incubated with sera of highly sensitized mice and stimulated with LamOVA or OVA. Beta‐hexosaminidase secretion after antigen stimulation is a measure of IgE cross‐linking. As shown in Figure 2C, a fivefold higher dose of LamOVA was required to activate IgE‐loaded RBL cells compared with OVA thus confirming hypoallergenicity of LamOVA.

### 3.4 Laminarin conjugation to OVA facilitates uptake by BMDCs and induces their activation

**Figure 3:**
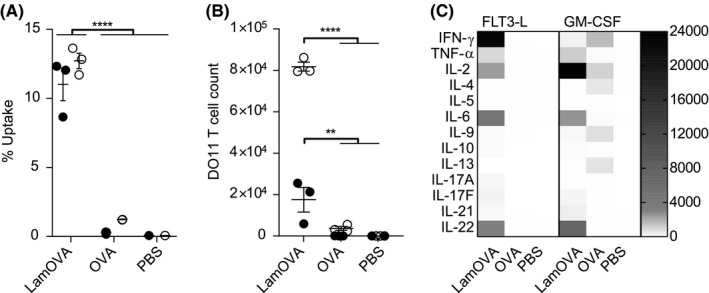
(A) Uptake of pHrodo‐labeled OVA or LamOVA conjugates by GM‐BMDCs (white circles) and FL‐BMDCs (black dots) was analyzed by flow cytometry after 24 hours. (B) Proliferation of OVA‐specific DO11.10 T cells after 5 days co‐culture with BMDCs and OVA or LamOVA. Data are shown as number of live, proliferating DO11.10 cells. (C) Cytokine concentration in BMDC‐T cell co‐culture supernatants in pg/mL. Means ± SEM and individual technical replicates (n = 3) are shown. Data were statistically analyzed by two‐way ANOVA followed by Tukey's post hoc test

Bone marrow cells were incubated with either hFLT3‐L (FL) or mGM‐CSF (GM) to generate BMDCs. Uptake of pHrodo‐labeled LamOVA was significantly enhanced compared to pHrodo‐OVA in both FL‐BMDCs and GM‐BMDCs (Figure 3A). LamOVA significantly activated BMDCs in a dose‐dependent manner, whereas unconjugated OVA and laminarin (used at doses equivalent to those present in LamOVA) did not induce CD86 expression even at the highest doses. GM‐BMDCs showed a higher base‐line expression of CD86 and responded more strongly to positive control stimulus LPS than to LamOVA (Figure S5A). In contrast, FL‐BMDCs had a more naïve phenotype and LamOVA induced an equivalent upregulation of CD86 compared with LPS (Figure S5B). In both cell types, LamOVA induced significantly higher CD86 expression than OVA, laminarin, and PBS stimulation (Figure S5AandB). LamOVA and laminarin stimulation significantly downregulated Dectin‐1 surface expression in GM‐BMDCs and FL‐BMDCs (Figure S5CandD), indicating binding of the beta‐glucan to its receptor. Interestingly, LPS activation also induced downregulation of Dectin‐1, which has already been observed in macrophages.^27^

LamOVA‐induced activation of BMDCs was associated with a robust, dose‐dependent cytokine and chemokine response (Figure S6). Compared to OVA and laminarin, LamOVA significantly upregulated all measured cytokines and chemokines in FL‐BMDCs (two‐way RM ANOVA, followed by Tukey's post hoc test). GM‐BMDCs displayed a much higher basal activation status than FL‐BMDCs as indicated by significantly higher levels of IL‐1α, IL‐1β, IFN‐γ, IL‐17A, IL‐6, IL‐27, CCL‐3, CCL‐4, CXCL10, CCL17, and CCL22 after 24 hours incubation without stimulus. CCL3, CCL4, CCL17, and CCL22 were especially elevated (37‐, 80‐, 138‐, and 101‐fold) compared to FL‐BMDCs. Despite this higher basal activation level, LamOVA significantly upregulated pro‐inflammatory mediators (TNF‐α, IFN‐β, IL‐6, IL‐23, CCL3, CCL4, CCL5, and CXCL10), and neutrophil attractants CXCL1 and CXCL5. In contrast, neither EndoFit™ OVA nor laminarin induced significant cytokine or chemokine secretion in either BMDC type.

### 3.5 LamOVA activated BMDCs are potent inducers of T‐cell responses

Laminarin conjugation to OVA significantly increased uptake and induced activation of BMDCs and secretion of pro‐inflammatory cytokines. These properties of LamOVA conjugate also resulted in enhanced stimulation of OVA‐specific naïve T cells co‐cultured with BMDCs. Both, FL‐ and GM‐BMDCs incubated with LamOVA significantly activated T‐cell proliferation and cytokine secretion (Figure 3B and C). GM‐BMDCs loaded with conjugates were more potent in inducing DO11.10 T‐cell proliferation than FL‐BMDCs, which may be due to their higher basal activation status (Figure S5). This is also supported by the fact that only GM‐BMDCs could induce activation of naïve T cells (Figure 3B) and significant secretion of cytokines (IFN‐γ and IL‐9, Figure 3C) in the presence of Endofit™ OVA. In both cell culture models, conjugates significantly induced IL‐2, IL‐6, and IL‐22 production (*P* < .0001, Figure 3C). Interestingly, LamOVA loaded FL‐ and GM‐BMDCS displayed a different TH‐polarizing potential as FL‐BMDCs mainly generated TH1 cells (IFN‐γ and TNF‐α, *P* < .0001 and *P* < .001), whereas GM‐BMDCs favored upregulation of IL‐2, which correlated with the higher T‐cell proliferation rate.

### 3.6 Epicutaneous immunization with LamOVA induces a strong antibody response

**Figure 4:**
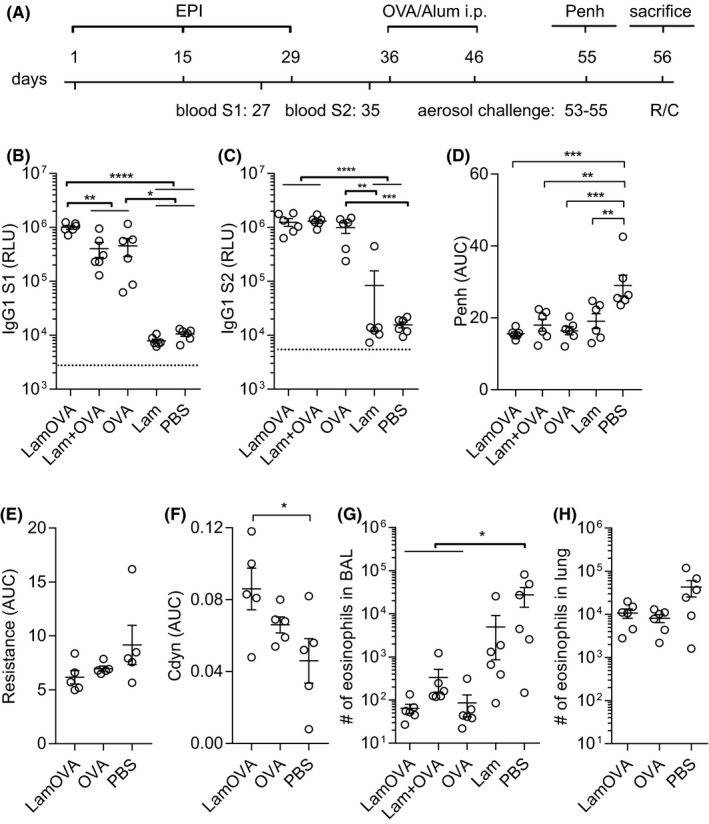
Prophylactic epicutaneous immunization via laser‐generated micropores (EPI). (A) Mice were immunized 3 times with LamOVA, OVA, a mix of OVA with laminarin (Lam + OVA), laminarin (Lam) or PBS followed by i.p. sensitization and aerosol challenge. Numbers indicate days. OVA‐specific serum IgG1 was measured after two (B, S1) or three (C, S2) immunizations at a serum dilution of 1:10 000. Data are shown as relative light units (RLU) of a luminometric ELISA (n = 6). Background is indicated by dotted line. Airway hyperresponsiveness was assessed by WBP (D, n = 6), as well as resistance (E, n = 5) and compliance (F, n = 5) measurements. Penh, resistance, and compliance data are shown as area under the curve (AUC) of a methacholine challenge dose‐response curve. Numbers of eosinophils in BAL (G, n = 6) or collagenase‐digested lung preparations (H, n = 6) were analyzed by flow cytometry

We have previously shown that epicutaneous immunization with mannan‐conjugated allergen via laser‐generated micropores (EPI) is considerably more immunogenic compared to immunization with unconjugated allergen.^15^ In a prophylactic immunization experiment (Figure 4A), we could confirm that EPI with LamOVA induces ~threefold higher IgG1 antibody titers compared with OVA after two immunizations (Figure 4B). This immune potentiating effect was dependent on the covalent linkage between OVA and laminarin since a mix of uncoupled OVA with laminarin did not enhance antibody responses. After a second booster immunization, all groups displayed similar antibody titers (Figure 4C). IgG2a levels were close to the detection limit (data not shown).

### 3.7 Preventive LamOVA immunizations reduce allergic lung inflammation and airway hyperresponsiveness

To investigate whether LamOVA‐EPI would protect from allergic sensitization and lung inflammation, vaccinated mice were sensitized by two i.p. injections of Endofit™ OVA/alum followed by three aerosol challenges.

Lung function was measured by whole‐body plethysmography (WBP) under methacholine challenge. Mice vaccinated with LamOVA showed the lowest Penh values, significantly lower compared to the sham‐treated control group (Figure 4D). The reduction of airway hyperresponsiveness (AHR) was confirmed in selected groups by invasive resistance (R) and compliance (Cdyn) measurement on the next day, confirming the WBP results (Figure 4E and F).

After R/C measurement, the cellular composition and cytokine content of bronchoalveolar lavage fluid (BALF) were analyzed. Preventive immunizations with OVA significantly reduced the number of eosinophils (Figure 4G) in BALF, with no differences between the OVA, LamOVA, and Laminarin + OVA groups. Eosinophil counts in whole lung tissue confirmed BALF analysis for OVA and LamOVA (Figure 4H). Eotaxin and IL‐5 concentration in BALF were similar in all groups and did not correlate with eosinophil number in BAL and lung tissue (data not shown).

In summary, our data show that despite their hypoallergenicity, conjugates are more potent in the production of OVA‐specific IgG antibodies and can prevent allergic sensitization and airway hyperresponsiveness. In a next step, we therefore evaluated safety and potency of LamOVA conjugates in a therapeutic mouse model of allergic asthma.

### 3.8 Epicutaneous immunotherapy with LamOVA induces blocking IgG and suppresses lung inflammation

**Figure 5:**
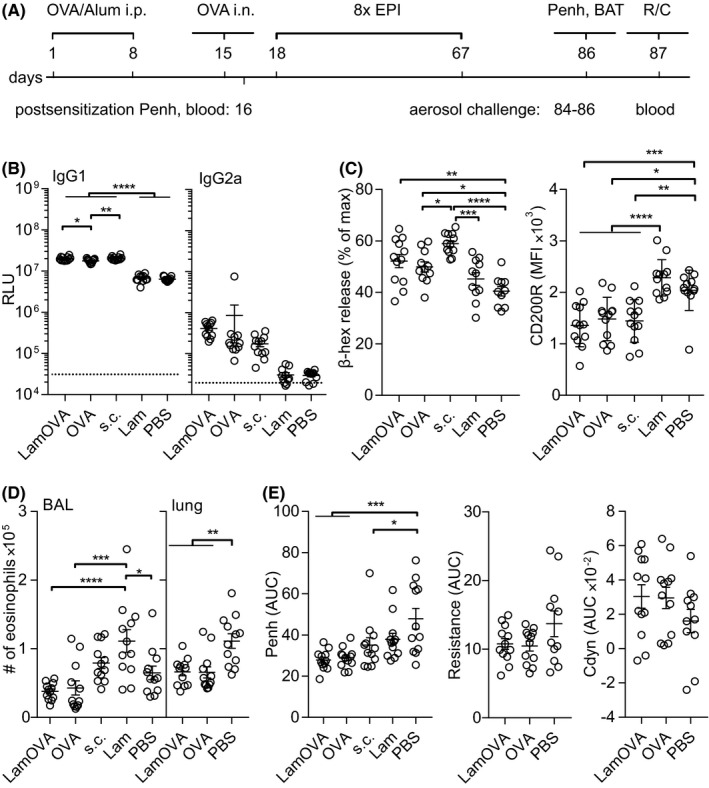
Therapeutic epicutaneous immunization via laser‐generated micropores (EPIT). (A) Mice were sensitized by two i.p. injections with OVA/alum and an intranasal challenge. After 8 treatments, either epicutaneously (LamOVA, OVA) or subcutaneously with OVA/alum (s.c.), mice were exposed 3 times to aerosolized OVA before sacrifice. Numbers indicate days. (B) OVA‐specific serum IgG1 and IgG2a at the timepoint of sacrifice were measured at a serum dilution of 1:10 000 and 1:400, respectively. Data are shown as relative light units (RLU) of a luminometric ELISA (n = 12). Background is indicated by dotted line. (C) Serum and cell‐bound IgE were analyzed by RBL assay (serum dilution 1:150) and BAT, respectively. (D) Number of eosinophils in BALF and lung tissue was analyzed by flow cytometry. (E) Airway hyperresponsiveness was assessed by WBP as well as resistance and compliance (Cdyn) measurements. Penh, resistance, and compliance data are shown as area under the curve (AUC) of a methacholine challenge dose‐response curve

In a therapeutic experiment, mice were sensitized by two i.p. injections of 10 µg Endofit™ OVA/alum followed by intranasal (i.n.) OVA challenge to induce lung inflammation. Based on WBP data, mice were stratified into five treatment groups with similar means and distribution of Penh. RBL assay confirmed that all animals had OVA‐specific IgE antibodies, however, treatment arms—by chance—showed slightly higher IgE levels before therapy compared with the control groups (Figure S7). Mice were treated 8 times epicutaneously (EPIT) with either LamOVA or OVA. As a positive control, mice were treated by subcutaneous injections of Endofit™ OVA together with alum (OVA s.c.). Controls were sham treated with either PBS or laminarin (Figure 5A).

LamOVA was equally potent in inducing IgG1 antibody production compared to s.c. injections together with alum. In contrast, EPIT with unconjugated OVA elicited significantly less antibodies. Treatment with conjugates showed a trend toward increased production of IgG2a antibodies (Figure 5B).

Subcutaneous immunotherapy with OVA significantly increased serum IgE levels compared to the PBS‐treated control group. The IgE increase after EPIT with LamOVA and OVA was less prominent compared to SCIT with OVA/alum (Figure 5C). It is known that AIT can temporarily increase serum IgE levels. However, more relevant is the blocking of basophil and mast cell activation induced by cross‐linking of long‐lived cell‐bound IgE. To test ex vivo blocking of basophil activation, blood samples were drawn 24 hours after the second exposure to aerosolized allergen and restimulated in vitro with OVA for 2 hours. Basophils from laminarin or PBS‐treated control groups showed high activation levels as indicated by the strong expression of CD200R. In contrast, basophils from OVA‐immunized groups showed significantly lower activation (Figure 5C). To test, whether the reduced basophil activation was due to the presence of blocking IgG, blood samples from treated mice were either washed or left untreated before in vitro restimulation with OVA. In the absence of autologous serum, basophils from all groups showed similar activation (Figure S8A), indicating the presence of comparable amounts of OVA‐specific IgE on the basophils. Thus, the IgG blocking capacity can be displayed as the ratio of basophil activation in the absence or presence of autologous serum. Correlating with the ELISA data, LamOVA EPIT group and OVA s.c. group showed the highest levels of blocking IgG (Figure S8B).

EPIT groups displayed the lowest number of eosinophils in BALF and significantly lower numbers in lung tissue compared with sham‐treated mice (Figure 5D). No significant differences in the BALF levels of IL‐4, IL‐5, IL‐13, and eotaxin were found (data not shown). OVA treatment significantly improved lung function measured by WBP and again the EPIT groups showed the lowest Penh values (Figure 5E). Though not statistically significant, a trend for reduced resistance and increased compliance in the EPIT groups was confirmed by invasive R/C measurement (Figure 5E).

Restimulation of splenocytes with OVA (10 µg/mL) induced increased expression of TH2 associated cytokines IL‐4, IL‐5, IL‐10, and IL‐13 but also IFN‐γ, IL‐2, IL‐6, and IL‐22 in the s.c. OVA group and surprisingly also in the group treated with laminarin alone. EPIT groups showed no such boost in cytokine responses and remained at similar levels compared with the PBS‐treated group (Figure S9). This effect was also seen on the level of transcription factor expression Tbet, GATA3, and RORγT. No difference in the number of FoxP3 + CD25+ Tregs was found in the spleens (Figure S10). In summary, s.c. injection with OVA/alum as well as treatment with laminarin boosted mainly TH2, but also TH1 and TH22 responses, whereas EPIT with OVA or LamOVA induced no increase in cytokine responses compared to the untreated group.

### 3.9 Conjugation of polysaccharide to OVA reduces local side effects in vivo

**Figure 6:**
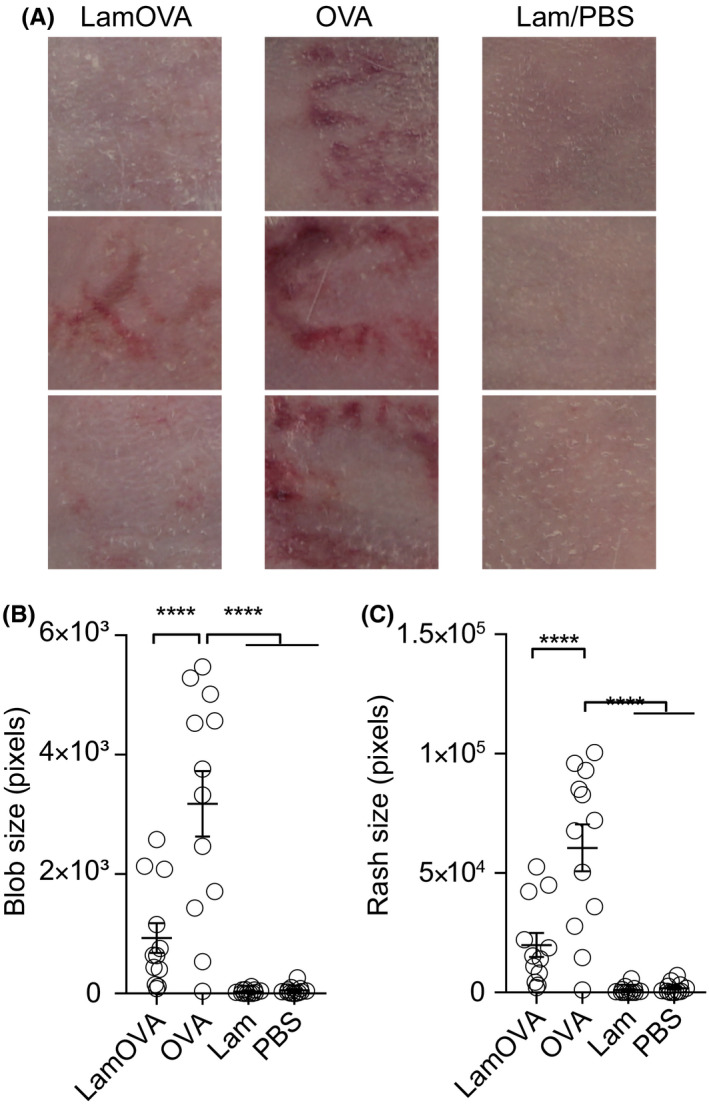
Skin erythema induced by EPIT (A) 24 hours after the 4th or 5th treatment, immunization sites were photographed and the sum of directly connected pixels of the segmented erythema (blob size, (B) and the total erythema size (C) were quantitated (n = 12). Panel A shows three representative animals from each group

In mice, local side effects are dependent on the used allergen. While in previous studies with recombinant Phl p 5^28^ or depigmented house dust mite extract,^29^ no local side effects were observed, application of OVA to the skin of sensitized mice led to significant skin erythema and scab formation (Figure 6A). We quantitated local skin reactions by calculating total erythema size and blob size (Figure 6B and C) and found that LamOVA immunizations induced significantly lower skin reactions, confirming the hypoallergenicity observed in vitro. The use of laser with PBS and laminarin alone did not induce any side effects, confirming the antigen‐specificity of the response.

## 4 DISCUSSION

Allergen‐specific immunotherapy (AIT) is the only disease‐modifying approach for treatment of allergies. However, classical subcutaneous immunotherapy (SCIT) suffers from low patient compliance due to exhaustive treatment protocols and occurrence of local and systemic adverse reactions. Therefore, more efficient and safe therapeutic concepts are urgently needed. Subcutaneous injections deliver antigen into the hypodermis, an adipose tissue rich in blood vessels, but sparsely populated by APCs. In contrast, epidermis and dermis are rich in APCs and (epi)cutaneous immunotherapy may result in enhanced immunogenicity and efficacy. Delivery of allergen to superficial skin layers avoids contact with blood vessels, decreasing the risk of systemic side effects.^30^, ^31^ Upon epicutaneous application, antigen is taken up by Langerhans and dendritic cells, which migrate to the lymph nodes for presentation of antigenic peptides to T cells. In a peanut allergy model, EPIT with Viaskin® occlusive patches led to formation of tolerogenic immune responses,^31^ demonstrating an important role of Langerhans cells in induction of regulatory T cells (Treg) in the context of skin immunization. Other APCs, which are able to generate Tregs, such as the CD11b + cDC2 subset, have been shown to reside in the dermis.^32^, ^33^ Though epicutaneous allergen delivery with occlusive patches has been shown to effectively induce tolerance, this approach affords high amounts of allergen to be daily applied over several years^4^ due to limited penetration of antigen through the outermost skin layer, the stratum corneum. In contrast, pretreatment of the skin for disruption of this tight barrier can improve immunogenicity of EPIT. This approach has been introduced in Europe during the 1950s and has only recently proven its potency to reduce allergic symptoms in clinical trials.^2^ In this work, we utilized an infrared laser (PLEASE® Professional) device for controlled and highly reproducible barrier disruption. In addition to physically breaching the stratum corneum for facilitated antigen delivery directly to skin resident APCs,^16^ laser microporation creates a proinflammatory cytokine and chemokine milieu, which boosts immunogenicity.^34^

Notably, Senti et al reported that allergen application onto barrier disrupted skin may result in local and systemic side effects,^2^ thus highlighting the need for hypoallergenic, yet immunogenic formulations. Coupling of carbohydrates to allergens represents an elegant approach to reduce the IgE binding capacity of the resulting neoglycoconjugates, while simultaneously targeting and stimulating dendritic cells. We have reported that mannan‐Bet v 1 conjugates (Bet‐MN) are particularly hypoallergenic and no longer induce basophil activation in vitro.^15^, ^35^ In a similar approach, Sirvent et al demonstrated hypoallergenicity of mannan allergoids using skin prick tests in allergic patients.^36^ In our current study, we found 4‐5 kD laminarin conjugates to be fivefold less allergenic in vitro (Figure 2B) and to induce significantly less local side effects in sensitized mice during therapy (Figure 6). Coupling of allergens to β‐glucan molecules with a higher molecular weight could potentially further decrease allergenicity as we have previously seen a >1000‐fold reduction of IgE cross‐linking capacity of 30‐40 kD mannan conjugates.^15^ However, a careful balance of protein/carbohydrate ratio has to be maintained, as a higher polysaccharide content in conjugates potentially decreases IgG production (unpublished observation).

Due to the low immunogenicity of existing allergy vaccines, AIT requires repetitive administrations of high allergen doses. More potent and controlled APC activation may be achieved via specific targeting of surface receptors,^6^ for example, C‐type lectin receptors.^37^ Here, we used laminarin to target Dectin‐1, which is expressed on various cell types including CD11b + dermal DCs and Langerhans cells in mice.^38^ We^15^, ^35^ and others^36^, ^39^ have previously reported enhanced uptake of neoglycoconjugates compared with unconjugated protein. In contrast to Sirvent et al, who observed that oxidation of mannan impaired uptake of conjugates,^36^ we have shown that mild oxidation of laminarin for allergen coupling does not influence its biological activity, as confirmed by its binding to Dectin‐1 (Figure 2A) and activation of BMDCs (Figures S5 and S6). We previously made similar observations for mannan^15^ and other carbohydrates.^35^ We employed two different in vitro models for studying antigen uptake, DC activation, and stimulation of naïve T cells, that is, Flt3L derived BMDCs and GM‐CSF derived BMDCs. While FL‐BMDCs more closely represent steady state naïve DCs, GM‐BMDCs rather resemble monocyte‐derived inflammatory DCs (and macrophages).^20^, ^21^ Both models are relevant for skin vaccine evaluation as they represent both, the early phase of the immune response, when naïve DCs first encounter an antigen, and the late phase response, when local inflammation (induced by adjuvants or laser microporation) attracts inflammatory DCs to the vaccination site. Indeed, GM‐BMDCs displayed a higher basal activation status in the absence of stimuli as indicated by increased levels of CD86 and Dectin‐1 expression (Figure S5) and enhanced secretion of cytokines and chemokines (Figure S6) compared to FL‐BMDCs. Laminarin is a β‐1,3‐glucan and, depending on the mode of uptake, can have pro‐ or anti‐inflammatory properties. For example, low molecular weight laminarins do not stimulate APCs,^25^ as Dectin‐1 activation occurs only after clustering of several units of the receptor.^40^ Hence, receptor oligomerization requires a specific size of β‐glucan complexes.^25^ Here, we demonstrate that (dialyzed) 4.5 kDa laminarin does not activate BMDCs in vitro, whereas laminarin‐OVA complexes are very potent in stimulating GM‐BMDCs as well as FL‐BMDCs. It has been shown that receptor internalization attenuates pro‐inflammatory signaling, whereas large particles such as zymosan cannot be internalized and induce strong pro‐inflammatory immune responses.^41^ Corroborating findings from Xie et al,^39^ our data indicate that LamOVA is taken up by receptor‐mediated phagocytosis as indicated by downregulation of surface Dectin‐1 expression (Figure S5) and transport into the endosome (Figure 3A). Yet, in contrast to data from others,^41^ we clearly observed the induction of inflammatory pathways in LamOVA stimulated BMDCs. In line with the BMDC activation status, only GM‐BMDCs could activate naïve OVA‐specific T cells without additional stimuli (Figure 3B and C). In contrast, BMDCs pulsed with LamOVA were potent activators of naïve T‐cell proliferation and cytokine secretion. As components of fungal cell walls, β‐1,3‐glucans are known to trigger TH1/TH17 adaptive immunity.^38^, ^42^ This could be confirmed in T cells stimulated with LamOVA loaded FL‐BMDCs, which secreted high levels of IFN‐γ, TNF‐α, and IL‐22, and low levels of IL‐17 (Figure 3C). Interestingly, although GM‐DCs induced stronger T‐cell proliferation and IL‐2 secretion, they were less potent in inducing TH1 cytokine secretion, which also correlates with their lower levels of IL‐12 secretion upon LamOVA stimulation compared to FL‐BMDCs (Figure S6).

We have recently demonstrated that application of allergens to laser‐microporated skin can induce humoral immune responses^16^, ^29^, ^43^ and that conjugation to mannan potentiates antibody responses.^15^ As shown previously for mannan, laminarin also boosted IgG1 responses threefold after EPI and covalent linkage of the carbohydrate to OVA was essential for this effect (Figure 4B). This can be explained by the multimeric state of laminarin, that is more potent in stimulating Dectin‐1 than soluble laminarin (Figures S5 and S6), the enhanced uptake due to receptor‐mediated endocytosis of the complexes (Figure 3A), and a potentially slower draining to SDLNs as previously observed for Bet‐mannan conjugates.^15^ A similar observation was made by Xie et al after s.c. immunization; however, they also included poly I:C as an adjuvant.^39^ In a prophylactic setting, EPI with OVA and LamOVA significantly suppressed induction of allergen‐induced lung inflammation. Although statistically not significant, LamOVA vaccinated animals showed lower lung resistance (Figure 4E) and higher dynamic compliance (Figure 4F) compared with OVA‐immunized mice. Thus, despite priming TH1/TH17 responses in vitro, LamOVA had no detrimental inflammatory effects in a prophylactic allergy model in vivo.

We have previously shown in a therapeutic mouse model of allergic lung inflammation that laser‐mediated EPIT with Phl p 5 or house dust mite extract can successfully reduce lung inflammation and improve lung function. Therapeutic efficacy was associated with induction of high levels of blocking IgG and a general downregulation of cytokine responses.^28^, ^29^ In the current study, we show for the first time the therapeutic efficacy of a hypoallergenic laminarin‐allergen conjugate. While EPIT with OVA was similarly effective in suppressing allergic lung inflammation and airway hyperresponsiveness compared with LamOVA, it was associated with severe local side effects (Figure 6), replicating findings from clinical trials with grass pollen extract.^2^ In contrast, LamOVA treated mice showed significantly ameliorated skin erythema. At the same time, conjugation to laminarin significantly boosted IgG1 (compared to OVA EPIT) to the same levels as achieved by s.c. injections together with alum (Figure 5A), while inducing lower levels of therapy‐associated IgE (Figure 5C). Furthermore, both EPIT groups showed lower levels of lung inflammation compared to s.c. treated mice (Figure 5D and E). Although LamOVA‐induced inflammation and TH1/TH17 polarization in vitro, EPIT with LamOVA did not result in enhanced inflammatory cytokine secretion in splenocytes from treated animals (Figure S9). In contrast, SCIT with OVA boosted TH2 responses, an early therapy effect that is well known in the clinics.^44^ Though we did not find increased numbers of FoxP3 + CD25+ regulatory T cells in the spleen after treatment, EPIT significantly reduced the number of activated T cells compared with SCIT (Figure S10). Taken together, this confirms our previous findings that EPIT via laser‐generated micropores can downregulate established allergic responses and mainly results in the induction of blocking IgG and a general suppression of cytokines^28^, ^29^ rather than immune deviation toward TH1 or TH17, even in the presence of an immunostimulatory Dectin‐1 agonist. While the first clinical studies of SCIT with mannan allergoids are currently under way (NCT02654223, NCT02661854), we believe that combining a C‐type lectin receptor‐based DC targeting approach with a method to deliver a hypoallergenic vaccine to upper skin layers rich in APCs is the key to patient friendly, safe and effective immunotherapy. Our current proof of concept study demonstrates that laser‐facilitated EPIT with β‐glucan‐allergen conjugates represents one such feasible approach.

## CONFLICTS OF INTEREST

The authors declare that they have no conflict of interest.

## AUTHOR CONTRIBUTIONS

EK, S. Scheiblhofer, IJ, HS, and RW performed in vivo mouse experiments. VS did in vitro BMDC experiments. M. Schubert performed NMR measurements and analysis. ED performed MST measurements and analyzed the data.TN, MS, and RB performed lung perfusions and prepared total lung digests. MG analyzed conjugates by DLS. S. Wildner and GG determined conjugate concentration by amino acid analysis. DJ, S. Schaller, and S. Winkler generated computational model and wrote the software for skin erythema analysis. RW, S. Scheiblhofer, and EK designed the study and wrote the manuscript. RW, EK, and JHH analyzed the data. S. Wildner, GG, and JHH critically revised the manuscript.

## Supporting information

Supplementary Material
